# Localization Preference of Antimicrobial Peptides on Liquid-Disordered Membrane Domains

**DOI:** 10.3389/fcell.2020.00350

**Published:** 2020-05-19

**Authors:** Juanjuan Su, Siewert J. Marrink, Manuel N. Melo

**Affiliations:** ^1^Molecular Dynamics Group, Groningen Biomolecular Sciences and Biotechnology Institute and Zernike Institute for Advanced Materials, University of Groningen, Groningen, Netherlands; ^2^Multiscale Modeling Lab, Instituto de Tecnologia Química e Biológica António Xavier, Universidade Nova de Lisboa, Oeiras, Portugal

**Keywords:** antimicrobial peptides, phase separation, liquid-ordered, liquid-disordered, pore, coarse-grain, molecular dynamics, enthalpy

## Abstract

We performed coarse-grained simulations of the antimicrobial peptides Magainin-2, BP100, MSI-103, and MSI-78 on a phase-separated membrane to study their preference for the different domains. All the peptides displayed a clear preference for the liquid-disordered (Ld) phase over the liquid-ordered (Lo) one. For BP100, MSI-103, and MSI-78 there was a further preference of the peptides for the domain interface. The peptides’ preference toward the disordered phase was shown to reflect a penalization of lipid–lipid interaction enthalpy in the Lo phase, when in the vicinity of peptides. Similar results were observed at the two studied concentrations, although Ld phase saturation at the higher concentration drove some of the peptide excess to the Lo phase. Magainin-2 and MSI-103 were found to dimerize, in agreement with available experimental data. Interestingly, at high concentrations of Magainin-2 toroidal pores spontaneously formed in the Ld phase. We performed additional simulations to characterize this phenomenon, which is likely related to Magainin-2’s membranolytic action.

## Introduction

Membrane lipid heterogeneity is crucial for various processes in living cells. Functions attributed to the membrane lipidome range from specific integral protein solvation (Contreras et al., 2011) to signaling (Forrester et al., 2004; Golub et al., 2004) to formation of spatial domains of different local composition (Simons and Toomre, 2000). Commonly used model systems are bilayers composed of ternary mixtures of cholesterol, saturated, and unsaturated lipids, which yield a rich phase behavior at physiological temperatures (Feigenson, 2006). Over a range of component concentrations these ternary mixtures laterally separate into a liquid-ordered (Lo) phase, enriched in the saturated lipid and cholesterol, and a liquid-disordered (Ld) one, enriched in the unsaturated lipid (Veatch and Keller, 2003; Marsh, 2009; Dewitt and Dunn, 2015). While such lipid segregation has not been observed at large scales in cellular membranes, the phase-separated ternary mixture model is still relevant, as there is plenty evidence of heterogeneity in lipid distribution, both in physiological membranes (Van Meer et al., 2008; Fadeel and Xue, 2009) and in complex models thereof (Ingólfsson et al., 2014). Understanding the interplay between membrane proteins and such heterogeneous surroundings is central to shedding light on the function of both proteins and the associated lipids.

An important class of proteins interacting with lipid membranes is formed by antimicrobial peptides (AMPs). Antimicrobial peptides are typically short cationic peptides and diverse both in sequence and structure (Epand and Vogel, 1999; Yeaman and Yount, 2003). The amphipathic α-helical structured AMPs are particularly abundant and widespread in nature (Oren and Shai, 1998). Their net cationicity and amphipathic character facilitates their incorporation into the negatively charged microbial membranes. The activity of AMPs has been observed to depend critically on the composition of target cell membranes. Many studies focus on model membranes containing negatively charged lipids, mimicking the bacterial membrane composition and allowing the characterization of the electrostatic component of the interactions between the cationic peptides and the host’s membranes (Epand et al., 2010; Polyansky et al., 2010; Wadhwani et al., 2012). At the same time, the disruption of cholesterol-containing homogeneous membranes by AMPs has attracted some interest since many of these peptides are able to kill fungi, protozoa, and enveloped viruses—all of which have sterol-rich membranes (Raghuraman and Chattopadhyay, 2004; Verly et al., 2008; Ramamoorthy et al., 2010). However, only a limited number of experimental studies have looked at AMPs interacting with phase-separated heterogeneous membranes (Brender et al., 2012; McHenry et al., 2012).

**FIGURE 1:**
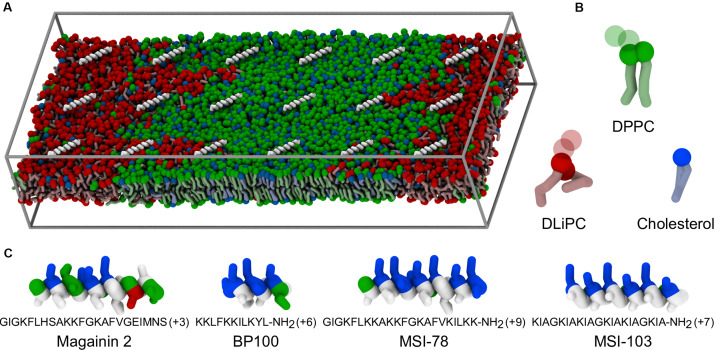
Coarse-grained representation of simulation box, peptides, and lipids. **(A)** Example of starting system configuration, showing the initial placement of low concentrations of MSI-78. The box size was of 47 nm × 19 nm × 10 nm. Components follow the representation scheme of the remaining panels, with the exceptions that, for clarity, peptides are shown only as white backbones and the charged particles of the phospholipid headgroups (phosphate and choline) are omitted; solvent is also not shown. **(B)** Representation of the coarse-grain structure of the used lipids. Full spheres highlight the polar coarse-grain particles (glycerol in the phospholipids, hydroxyl moiety in cholesterol); translucent spheres indicate the charged phospholipid particles not depicted in **(A)**. **(C)** Coarse-grain structure of the studied α-helical peptides, colored according to residue polarity/charge (apolar: white, polar: green, acidic: red, basic: blue) and highlighting their amphipathic nature.

In this work, we use molecular dynamics (MD) simulations to study the interactions of four AMPs—Magainin-2, BP100, MSI-103, and MSI-78—with phase-separated model membranes composed of cholesterol, a saturated lipid (di-palmitoyl phosphatidylcholine; DPPC), and a polyunsaturated lipid (di-linoleyl phosphatidylcholine; DLiPC). The four peptides were selected to represent different sizes and charge densities, although all four share the same α-helical secondary structure and cationic nature, both typical of AMPs (Figure 1). Magainin-2 (23 residues) has a low net charge (+3), while MSI-78 has a similar length (22 residues) but a much higher cationic charge (+9); MSI-78 has also been experimentally investigated by McHenry et al. (2012) on phase-separated lipid membranes. BP100 has the highest charge density (+6 over 11 residues). MSI-103 (+7, 21 residues) is a somewhat intermediate example, and also the only one of the peptides that has an entirely synthetic origin (Strandberg et al., 2008), all others being either naturally occurring AMPs or inspired by AMPs that are.

We use the Martini coarse-grained force field (Marrink et al., 2007), widely used in simulations of phase-separating bilayers (Risselada and Marrink, 2008; Bennett and Tieleman, 2013). Previous simulation studies using this model have shown a preference of other compounds—transmembrane proteins (Schäfer et al., 2011; Domański et al., 2012), sugars (Moiset et al., 2014), aliphatics (Barnoud et al., 2014; Rossi et al., 2014), and drugs (Muddana et al., 2012)—to partition into the Ld phase, or to adsorb at the domain boundaries, sometimes leading to domain remodeling. Here, our aim is to ascertain the phase preference of AMPs and to explain the driving forces behind it.

## Materials and Methods

### Force Field and Simulated Systems

In this work, we employed Martini version 2.2 for our lipids, cholesterol, and peptide parameters (Marrink et al., 2007; Monticelli et al., 2008; de Jong et al., 2013; Melo et al., 2015). The representation of the simulation box, peptides and lipids is shown in Figure 1. We used the Avogadro software (Hanwell et al., 2012) to build the initial atomistic structures of each peptide, assuming an entirely α-helical secondary structure. The coarse-grained Martini structures were obtained using the *martinize.py* script. The simulated bilayers were composed of DPPC, DLiPC, and cholesterol at a 42:28:30 ratio, for a total of 3628 lipids. A membrane patch was first equilibrated in water for 2.3 μs until equilibrium Lo/Ld phase separation was reached, following the pioneering work of Risselada and Marrink (2008). A rectangular patch shape of large aspect ratio was chosen so that phase domains could easily become continuous with themselves across the periodicity in the *y* direction, thus reducing the phase interface line tension. Each AMP was added to a separate copy of this patch, placed at the surface of the membrane. Two peptide-to-lipid (P:L) ratios were employed: 1:200 and 1:20. All systems were made charge neutral by addition of the appropriate amount of chloride ions. At the highest concentration case, peptides were added to both sides of the membrane, in equal numbers, to prevent bilayer disruption due to sheer tension mismatch. Prior to production runs, peptide orientation and depth were equilibrated for at least 200 ns under the following adsorption protocol (adapted from Su et al., 2018): first and last backbone particles (also called “beads”) of each peptide were restrained in the *xy* plane with a harmonic potential of 3000 kJ mol^–1^ nm^–2^ to prevent lateral diffusion and untimely peptide–peptide association. A harmonic restraint in *z* (5000 kJ mol^–1^ nm^–2^) was further applied on the peptides, pulling them to a distance of 1.4 nm from the bilayer center, and preventing dissociation into the aqueous phase. This setup allowed the peptides to rotate parallel to the bilayer plane to optimally face the bilayer, without getting trapped in pre-equilibrium aggregates. Production runs then proceeded without any restraint on the peptides for a minimum of 60 μs, with configurations saved for analysis every 30 ns.

### Simulation Parameters

All the simulations were performed using the GROMACS software (Hess et al., 2008) version 4.6.7 or version 5.1 when the use of flat-bottom restraining potentials was needed. Periodic boundary conditions were used. The temperature was coupled (coupling time 1.0 ps) to T = 295 K, using the Berendsen thermostat (Berendsen et al., 1984). The pressure was coupled using the Berendsen barostat (Berendsen et al., 1984) (coupling time of 0.5 ps and compressibility of 4.5 × 10^–5^), using a semi-isotropic coupling scheme in which the lateral and perpendicular pressures were coupled independently at 1 bar, corresponding to a tension-free state of the membrane. Non-bonded interactions were computed as Lennard-Jones (LJ) potentials, switched to zero from 0.9 to 1.2 nm (pair-list update frequency of once per 10 steps). Electrostatics were calculated as Coulombic interactions with an implicit dielectric screening constant of 15, shifted to zero from 0 nm to the same 1.2 nm cutoff. A time step of 30 fs was used.

### Buckling Restraints

Membrane buckling can be expected to occur, independently of peptide presence, in these systems with large box vectors. To dampen it, one glycerol bead of each lipid was position-restrained in the *z* direction with a weak (300 kJ mol^– 1^ nm^– 2^) quadratic potential. This strategy, also successfully applied to more complex membranes (Ingólfsson et al., 2014), allows the use of a box size of smaller height without the risk of direct interaction between peptides and lipids of adjacent periodic images in *z*. In a reference simulation, we used as an alternative a flat-bottomed potential restraint of 1000 kJ mol^–1^ nm^–2^, confining glycerol moieties to a *xy* slab of defined (4 nm) vertical thickness. This potential, available since GROMACS version 5.0, is harmonic at the slab edges but zero throughout. It therefore only acts on particles leaving the slab region, allowing the headgroups to move freely toward the bilayer core and even flip-flop.

### Analysis

The analyses focus on the last 20 μs of each production simulation and extensively employ the NumPy (van der Walt et al., 2011), scikit-learn (Pedregosa et al., 2012), scikit-image (van der Walt et al., 2014), and MDAnalysis (Michaud-Agrawal et al., 2011; Gowers et al., 2016) Python packages. Values are reported as averages over those 20 μs, with 95% confidence intervals estimated using a bootstrap procedure with 1000 resamplings. Underestimation of uncertainty due to time-correlated data was corrected for by bootstrap-resampling a lower number of datapoints, proportionally to the integrated autocorrelation time of the observation (expressed in number of frames). As an example: for the 666 data points in the analyzed 20 µs at a rate of 30 ns/frame, for an observation with an integrated autocorrelation time of 2 frames, bootstrap resamplings were only done for 333 data points. All the measured values on which analysis was performed are included as a Supplementary Data archive. The Python code used to generate such datasets is included in the same archive.

### Phase Assignment and Peptide Depth

**FIGURE 2:**
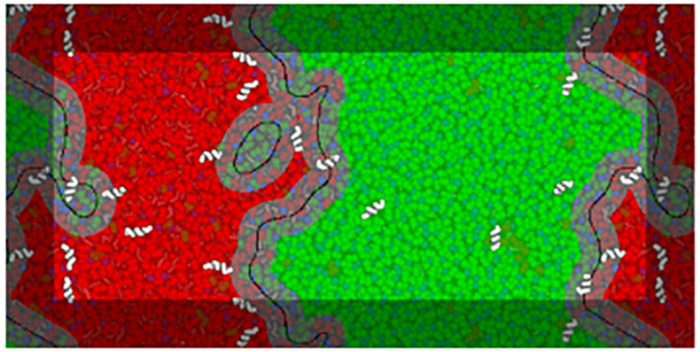
Example of interface analysis on a frame of a BP100 simulation at low peptide concentrations (matching the respective panel in Figure 3). A visualization of the analysis is overlaid on a representation of the system configuration, peptides being shown above the overlay for clarity. Black lines trace the identified interface phase boundaries, with a 1.5 nm contour—the interface region—in gray. The Lo and Ld domains are shown in translucent green and red, respectively. The interface lines are depicted with a thickness proportional to the width of the phase assignment grid spacing (1 Å).

To identify different domains, a phase-assignment algorithm (Jefferys et al., 2014) was implemented. In this algorithm lipid positions were flattened in *z* and smoothed using a Gaussian kernel with a 1.0 nm standard deviation. The first lipid tail beads (particles C1A and C1B) of DPPC and DLiPC were used to represent each species. The smoothed signal was sampled on a point grid of 1 × 1 Å spacing. The phase domain interfaces were determined by subtracting the DPPC signal from the DLiPC signal and running the result through the scikit-image Canny edge-detection filter. Analysis edge effects due to box periodicity were avoided by including beads of the neighboring periodic images and extending the phase assignment region by 2 nm in each direction. Figure 2 schematizes the analysis of a system in the presence of low concentrations of BP100.

To quantify the domain preference of the AMPs, peptide backbone beads were assigned to each of three possible regions: Ld, Lo, or interface, based on their *z*-flattened *xy* position. Beads were considered interfacial if within a 1.5 nm radius of an interface grid point. This radius was chosen for corresponding to a phosphate–phosphate distance that includes at least the first two neighbor shells; robustness of results to this choice was tested with radii from 1.2 to 2.0 nm (Supplementary Figure 4). Remaining backbone beads were considered to be in the Ld (resp. Lo) region if they were closest to a cell of that type. The counts of assigned backbone beads were normalized by the expected counts in each respective region given a random peptide distribution, which amounts to a normalization by region area fraction and global peptide density. The resulting value represents the ratio of enrichment of each region relative to a random peptide distribution.

The depth of peptides inserted into the membrane was measured as the *z*-distance of backbone beads to the center-of-mass of the phosphate beads of lipids within a 2 nm *xy* radius.

### Enthalpy Decomposition

The GROMACS package allows for the decomposition of interaction energies between defined groups. These groups, however, cannot be dynamically updated throughout the simulation, making the GROMACS tools unsuited to decomposing energies based on phase location criteria. To this end we implemented the Martini force-field nonbonded energy functions in Python and were then able to calculate nonbonded interactions between dynamic groups. We recreated the coulombic and LJ interaction potentials as per Supplementary Equation 1 through 6. Potential shapes were confirmed to be accurate by comparison to the debug output of the GROMACS mdrun program, which allows the inspection of the potential shapes used during simulation in tabulated numerical form. We focused energy analysis on the interactions between peptides, lipids, and solvent, discriminating the location of each peptide residue (based on the backbone bead location of said residue) as either Ld, Lo, or interfacial. As a further test of the accurate reproduction of nonbonded energies by our in-house code, a 20-frame trajectory segment was analyzed and the phase-discriminated peptide–lipid and peptide–solvent nonbonded energies were summed for the three considered phases. The resulting sum compared very well to the non-discriminated values calculated by the GROMACS package, with a maximum absolute difference of 8 × 10^–3^ kJ mol^–1^ and a maximum relative difference of 3 ppm, likely attributable to the differences in order of floating point operations (Supplementary Figure 5). The decomposition code is included in the Supplementary Data, archive alongside the dataset produced with it and scripts for further processing into the data presented here.

To estimate the enthalpic impact of peptide adsorption on lipid interactions a second discrimination was performed: lipids were classified as either Ld, interfacial, or Lo (depending on the closest phase-assignment grid point to the lipid’s phosphate bead), and as near to or far from a peptide depending on whether any of the lipid’s particles are within the 1.2 nm interaction cutoff of any peptide particles. Interfacial lipids were not considered for purposes of energy discrimination. Energies were calculated between each of the four discriminated groups (Ld/Lo, near/far) and the non-discriminated groups of all lipids, peptides, and solvent. Energies were normalized by the number of near/far lipid molecules and by the number of peptide residues in the respective Ld/Lo region—see more details in the accompanying Supplementary Figure 2.

Enthalpy decomposition was processed independently for each leaflet. Because cholesterol molecules can undergo flip-flop in the simulation’s timescale, they further had to be frame-by-frame dynamically assigned to each leaflet; a leaflet’s cholesterols were defined to be those with their hydroxyl bead within 2 nm of any of the leaflet’s lipid phosphate beads.

### Cluster Size Determination

To quantify oligomerization order, a 0.6 nm cutoff-based neighborhood graph between backbone beads was constructed. However, to still be able to have meaningful results at high peptide densities, where chance contacts are frequent, the following rules were employed: (i) two peptides are in contact, and establish a cluster, if at least three of each peptide’s backbone beads are neighbors to any of the other peptide’s backbone beads; (ii) a peptide is part of a cluster if at least three of its backbone beads are neighbors to at least three backbone beads of the cluster’s peptides (regardless if the contacted beads are all from a single peptide in the cluster or from multiple ones); and (iii) two clusters with peptides in common are considered a single cluster. These criteria were tuned to match visual assignments in representative cases, by favoring the counting of side-by-side peptide oligomerization as cluster-forming relative to weaker-bound situations (such as when two peptides meet in a T formation). Cluster size distributions are presented not as the number of clusters of each order, which visually inflates the contribution of low-order clusters, but as the fraction of total peptides taking part in clusters of each order.

## Results

### Peptide Oligomerization

**FIGURE 3:**
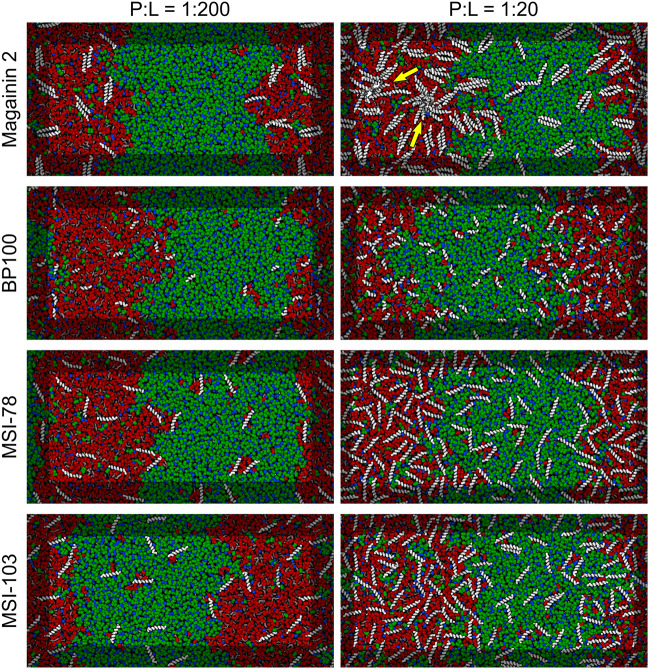
Partitioning behavior of antimicrobial peptides. Snapshots of simulations at 60 μs at low (P:L = 1:200, **left column**) and high (P:L = 1:20, **right column**) peptide concentrations. DPPC, DLiPC, and cholesterol are colored in green, red, and blue, respectively. Backbones of each peptide are shown in white (peptide names are indicated at the left of the panels). Two star-shaped aggregates can be seen for Magainin-2 in the Ld phase where pores were observed to form (indicated by yellow arrows).

**FIGURE 4:**
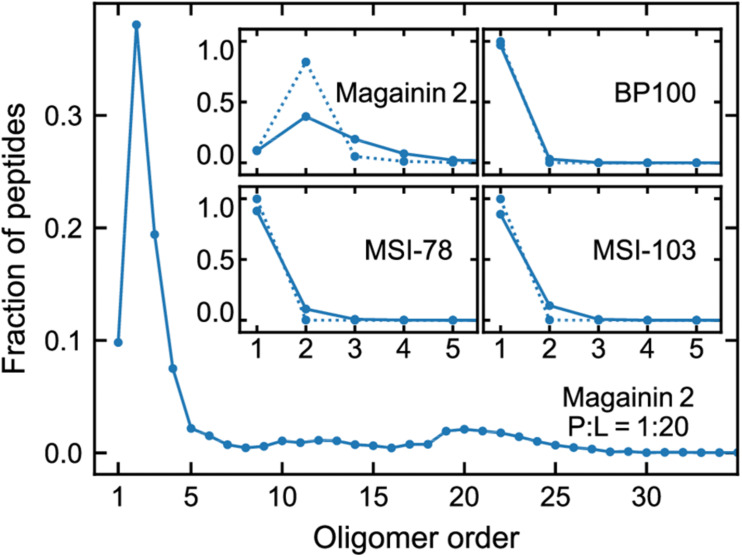
Oligomerization preference of each peptide, analyzed over the last 20 µs of simulation and expressed as the fraction of the total peptide per oligomer order. Main plot: distribution for Magainin-2 at high (P:L = 1:20) concentrations. Insets: distributions for all the peptides, at P:L ratios of 1:200 (dotted line) and 1:20 (full line), with plots focused on low oligomerization numbers.

Figure 3 shows the lateral organization of the peptides after 60 μs simulation time, at 1:200 and 1:20 P:L ratios. Focusing at the level of peptide–peptide interactions, the oligomerization behavior clearly differs between Magainin-2—mostly a dimer—and the other AMPs—mostly monomers. The oligomerization state is quantified in Figure 4, showing a much broader size distribution for Magainin-2, with a peak for the dimeric state regardless of concentration. This is in good agreement with the experimental observation of Magainin-2 dimer formation (Mukai et al., 2002; Wakamatsu et al., 2002). We observed MSI-103 to also dimerize, transiently and to a low extent (12%) at high concentrations (Figures 3F, 4–inset), in line with experimental evidence of environment-dependent MSI-103 aggregation (Toke et al., 2004). MSI-78 and BP100 have an even lower dimerization propensity, reaching only 9% and 3% rates of dimerization, respectively, at high concentrations. Although there is no direct experimental evidence that this is indeed their preferred oligomerization state, MSI-78 and BP100 have cationic charge densities higher than those of either Magainin-2 or the weakly-dimerizing MSI-103. BP100 or MSI-78 aggregation is therefore an unlikely event, consistent with our observations, and supported by the overall agreement of simulated Magainin-2/MSI-103 oligomerization with experimental data. Because AMP oligomerization is dependent, at least in part, on charge interactions, the observed agreement with experimental behavior validates our use of Martini’s coarse electrostatics and of a simplified ionic environment with only neutralizing ions.

### Peptide Phase Localization

**FIGURE 5:**
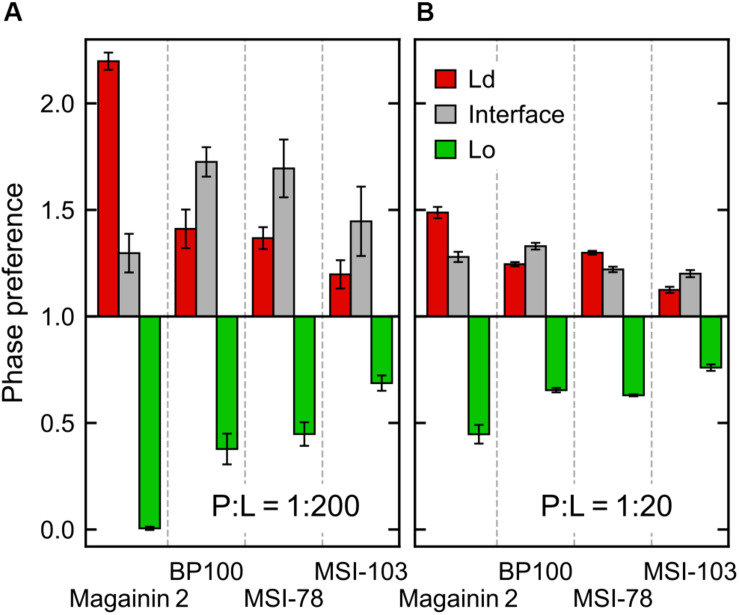
Ratio of peptide enrichment in each region relative to a random distribution. **(A)** At low (P:L = 1:200) peptide concentration; and **(B)** at high (P:L = 1:20) peptide concentration.

According to the experimental data of McHenry et al. (2012), MSI-78 has a preference for the Ld phase. Our simulations display the same behavior, as can be inferred from Figure 3. In fact, one can observe that all four AMPs prefer the Ld phase. The affinity for the disordered domain does not seem to be the same for all peptides, though. Magainin-2 is virtually absent from the ordered phase at low concentrations, whereas MSI-103 seems to distribute more homogeneously. Figure 5 shows the quantification of these observations over the last 20 μs of each trajectory. Indeed, at low concentrations there is a preference of all peptides for the Ld phase over the Lo one. It is the interfacial region, however, that is the most enriched, for all peptides but Magainin-2.

At high concentrations, and for all peptides, the enrichment ratio of each region is brought closer to 1 (Figure 5B). This seems to indicate that the preferred regions (interface and/or Ld) get saturated and the excess peptide is forced into the remaining phases. In the particular case of MSI-78 the preference for the interface region over the Ld phase becomes reduced. This may be related to some degree of lowering of line tension by MSI-78, as also visible in Figure 3 and in the relative phase areas, discussed below.

The above phase preference observations are mostly independent of the chosen radius for interface definition, but the preference values themselves are not (Supplementary Figure 3): analysis at a narrower radius yields a higher interfacial preference, presumably due to less averaging being performed over the depleted region of the Lo phase.

At low AMP concentrations phase areas are mostly unchanged by peptide type, with relative values around 33%, 20%, and 47% for Ld, interface, and Lo phases, respectively (see the detailed data in Supplementary Figure 3). High peptide concentrations bring about area changes only for the cases of MSI-78 and MSI-103. For both peptides there is an increase of the interface area, which reflects a lowering of the Ld–Lo line tension also visible in Figure 3. Interestingly, under MSI-78 the interface grows mostly at the expense of the Lo phase, whereas for MSI-103 it grows at the expense of both Ld and Lo phases (Supplementary Figure 4).

### Driving Forces

**FIGURE 6:**
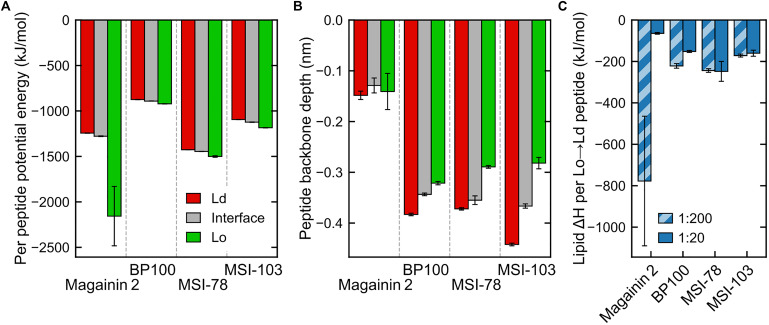
Interaction energies and penetration depths across domains (for panels **(A,B)** only data for the less concentrated systems, P:L = 1:200, is shown—see Supplementary Figure 1 for a side-by-side comparison with measured energies/depths at both peptide concentrations). **(A)** Total peptide nonbonded interaction potential energy in each region, averaged by the number of peptides in that region (intrapeptide nonbonded contributions excluded). **(B)** Peptide backbone in-depth position, expressed as the z-position difference to the average of nearby phosphate beads, for each region. **(C)** Difference in global lipid–lipid potential energy per peptide moved from the Lo region to the Ld region, at both concentrations. The very low numbers of Magainin-2 visiting the Lo phase at low concentrations (see Figure 5) introduce a large uncertainty in the measured value.

The concentration of peptides in any given region is clearly entropically unfavorable. We set out to identify the enthalpic forces driving this phenomenon. In case of transmembrane helices, it has been shown (Schäfer et al., 2011) that the main driving force for sorting of the peptides to the Ld phase is caused by changes in lipid–lipid enthalpy: the transmembranar peptides are essentially incompatible with the ordered nature of the Lo phase and disturb the tight packing of saturated lipids and cholesterol; this leads to an enthalpic driving force for sorting into the Ld domains. In case of the surface-adsorbed AMPs, one might expect little difference in the enthalpy of peptide interaction with either phase because DPPC and DLiPC have identical headgroups. However, a direct inspection of the peptide interaction enthalpies does show a difference between Ld-adsorbed and Lo-adsorbed peptide potential energy (Figure 6A). Furthermore, and contrary to what would be expected from the peptide localization preferences, it is the Lo-adsorbed state that is the most energetically favorable—even if the peptides interact with shallower adsorptions (Figure 6B). We can rationalize this enthalpic preference as likely stemming from the increased density of peptide–lipid interactions in the more compact Lo-phase, but this does not explain the peptide preference for the Ld/interface regions over Lo. We then monitored the effect of peptide presence on lipid–lipid interactions.

Energy discrimination, plotted in detail in Supplementary Figure 2, shows that the presence of peptides mostly leads to more favorable global interactions by nearby lipids, regardless of phase. In other words, even though peptides prefer the Ld phase, lipids would enthalpically rather have a peptide adsorbed on the Lo phase than to have no peptide at all (exceptions are the cases of Magainin-2 at low concentrations—for which there is also a large a measured error—and BP100, also at low concentrations). A main contributor to this is the aforementioned favorable peptide–lipid interactions. However, peptide adsorption can be seen to always come at a cost to lipid–lipid interactions: comparison between lipids in-contact and not-in-contact with peptides shows that in both phases lipid–lipid interactions become significantly weakened in the presence of peptides, and more so in the Lo phase. Lipid–solvent interactions seem to be largely insensitive to peptide presence.

These observations point to the effect that makes it overall enthalpically advantageous for a peptide to move from the Lo phase to the Ld phase: it makes lipid–lipid interactions more favorable in the Lo phase by an amount of energy that more than compensates for both the weaker lipid–lipid interactions (Supplementary Figure 2) and weaker peptide–lipid interactions (Figure 6A) in the Ld phase. This then yields the negative enthalpies in Figure 6C in the order of hundreds of kJ mol^–1^ per peptide that justify why the peptides can accumulate so clearly against the entropic tendency. Interestingly, the impact of lipid–lipid interactions occurs even though the AMPs are mostly adsorbed on the bilayer surface, and not transmembranar as previously studied transmembrane helices (Schäfer et al., 2011; Lin and Gorfe, 2019) that become phase-sorted by similar driving forces.

The argument of enthalpic competition between the Lo-lipid packing and peptide adsorption further explains why the peptides display a preference for the phase interface over even the Ld phase: at the interface the energetic cost of disrupting Lo-lipid packing is already partly paid for; peptides can then interact with Lo-lipids to some extent, and therefore have a lower global interaction energy, without incurring in the lipid–lipid energetic penalty that drives them away from the Lo phase.

Higher AMP concentrations, for the most part, did not bring about changes in the energetic driving forces. Enthalpy analysis indicates that the increase of AMP accumulation in the Lo phase with concentration results indeed from overcrowding of the Ld phase: overall peptide interactions in the Ld phase become more unfavorable at high concentrations, when compared to Lo (Supplementary Figure 1C).

### Magainin-2 Pore Formation at High Concentrations

At high P:L ratios two Magainin-2 pores were observed to spontaneously form in the Ld phase (Figure 3). From the peptide’s mechanistic point of view, this is an expected event (Ludtke et al., 1996). However, it is a significant observation with the Martini model because coarse-grain simulation of AMP pore formation often requires promoting the insertion step—either by multiscale approaches (Rzepiela et al., 2010), the self-assembly of membranes with peptides (Cruz et al., 2013; Balatti et al., 2017), or the pre-insertion of peptides (Woo and Wallqvist, 2011; Balatti et al., 2017). Successful descriptions of AMP insertion and pore formation from unbiased atomistic simulations (Leontiadou et al., 2006; Wang et al., 2016) show that these are observable in the microsecond scale, and therefore reachable by the faster Martini model. The reasons behind the difficulty of simulating pore phenomena with Martini are not entirely clear, although it has been pointed out that defects hardly form in Martini membranes, even during lipid flip-flop or crossing by polar moieties (Bennett and Tieleman, 2011). Indeed, Martini membranes covered with Magainin-2 will sooner buckle and bud than form pores (Woo and Wallqvist, 2011). In our simulations at high peptide densities membrane buckling was prevented by the application of a restraining potential in the *z* direction on the lipid headgroups; tension was further balanced by having equal amounts of peptide on each leaflet. Under this light, we believe that the extreme Magainin-2-induced buckling observed by others (Woo and Wallqvist, 2011) is probably a consequence of the reluctance of the Martini model for pore formation: once we removed membrane buckling as an energy outlet, we were able to readily observe pore formation. Furthermore, preventing a large membrane from naturally buckling may introduce additional pore-inducing tension. This factor alone, however, cannot explain why pore formation was only observed for Magainin-2 when all AMPs were simulated under buckling restraints.

Another possibly relevant aspect of how the observed pores formed has to do with (i) the careful pre-simulation peptide placement/adsorption protocol (Su et al., 2018), which prevented untimely peptide association and (ii) the gradual and spontaneous concentration of peptides in the Ld phase, which prevented the artifactual introduction of tension when peptides are forced together with unfavorable contacts.

**FIGURE 7:**
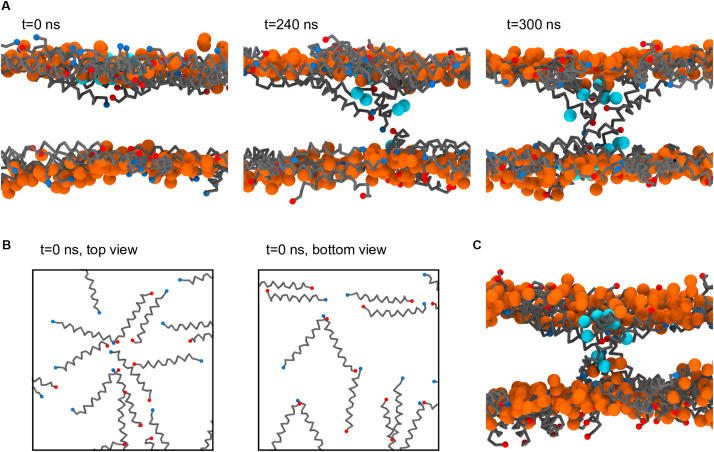
Process of Magainin-2 pore formation. **(A)** Three side-view snapshots depicting peptide internalization from the top leaflet, contact with the bottom leaflet, and establishment of a stable pore (see the main text for more detail). Label times are relative to the beginning of internalization, with *t* = 0 corresponding to 7.8 μs simulation time; for this system buckling was prevented by restraining vertical headgroup movement; the view at *t* = 300 ns is rotated by 180° around the *z* axis relative to the other two panels. **(B)** Top and bottom leaflet views of the peptide organization at *t* = 0, for the same system as in **(A)**. **(C)** Pore structure obtained in a membrane held flat by a flat-bottomed potential, as opposed to the headgroup restraints in **(A,B)**. Peptide backbones are depicted in gray, with blue and red N and C termini, respectively; lipids are shown only as their phosphate bead, in orange (these are mostly, but not entirely, DLiPC lipids, since the pores formed in the Ld phase); the water beads closest to the pore core are shown in cyan; for clarity, all other system components were hidden, and in **(B)** the phosphate beads were also not shown.

Figure 7A depicts a step-by-step formation of one of the Magainin-2 pores. Preceding pore formation Magainin-2 dimers, mostly antiparallel, assemble in one of the leaflets in a roughly radial fashion (Figure 7B). For both pores, formation involved the incursion of top leaflet peptides into the bottom leaflet. Two-to-three peptides, of mixed orientation and of different dimers, penetrate simultaneously end-first into the bilayer core (the mixed N- and C-terminal orientations presumably stabilizing the terminal charges), dragging water beads along. Peptides from the bottom leaflet then tilt inward to meet the incoming top-leaflet peptides, after which the pore is established. In both cases, after the initial radial aggregation, the process was quite fast, with the first peptide becoming transmembranar within 300 ns. Once inserted, peptide organization is tilted and no longer dimeric. The pore structure is also dynamic, with peptides frequently exchanging between the adsorbed and internalized states. However, no peptides were observed to fully translocate and adopt the adsorbed configuration in the opposing leaflet; there were, at most, cases where transmembrane peptides cross the membrane all the way and become anchored closer to the opposing leaflet than the starting leaflet. Regardless of peptide dynamics, once formed the pores themselves were stable for the remainder of the run (extended over 60 μs after pore formation) and were able to conduct water.

As stated, the observation of Magainin-2 pore formation seems to have been facilitated by the restriction of lipid headgroup movement in the out-of-membrane-plane direction. Naturally, this raises concern about the significance of the pore structure since such potentials prevent the lipid headgroups from accompanying the internalizing peptides, as is expected to occur for toroidal pore models (Ludtke et al., 1996; Leontiadou et al., 2006). To test the influence of this bias we employed a different method to restrict membrane buckling, namely a flat-bottomed potential (see Materials and Methods). Under the less biasing flat-bottomed potential two Magainin-2 pores were also observed to form (Figure 7C), confirming that it is not the specific restriction of headgroup movement that is promoting pore formation. The final structure of these pores is quite similar to the pores obtained with the headgroup restraints, with peptides inserted in a tilted, monomeric fashion. Incidentally, though the membrane is allowed to become somewhat distorted near the pores when using the flat-bottomed potential, headgroups still do not completely follow the peptides into the bilayer core. This behavior may be connected to the lack of membrane defects under the Martini model during lipid flip-flop (Bennett and Tieleman, 2011). Similar pore formation occurred for both types of potentials, even though flat-bottomed potentials controlled buckling without imposing nearly as much bias as harmonic position restraints. System restraining energies, before pore formation, were of 38.9 ± 0.5 kJ mol^–1^ for the flat-bottomed case vs. 3106.4 ± 5.7 kJ mol^–1^ for the harmonic case. Upon pore formation flat-bottomed energies remained unchanged (38.5 ± 0.6 kJ mol^–1^) but harmonic ones dropped to 3068.8 ± 2.0 kJ mol^–1^, suggesting that in this case lipid restraints may drive pore formation more directly.

## Discussion

In this work we were able to observe and assign the molecular bases for AMP preference for disordered phases. This extends and complements available experimental studies, in which a preference of AMPs for the Ld phase could be inferred, but only from indirect evidence (McHenry et al., 2012). Our findings show that AMPs disrupt lipid–lipid interactions in both phases, but mostly in the Lo phase. This ultimately causes the AMPs to locate in the Ld phase, even though the AMPs themselves do establish more favorable interactions in the Lo phase. The observed further preference of AMPs for the phase interface is a corollary of these energetic considerations. The relative depletion of Magainin-2 and MSI-78 at the interface, on the other hand, were interesting observations and merit further research. The choice of studied peptides and lipid mixture focused the scope of our conclusions on α-helical AMPs, on a particular phase separation condition. Future work on AMPs of different predominant secondary structure, on membrane with different degrees of lipid saturation, could shed light on other aspects of phase preference.

In addition to Ld phase preference, in the case of Magainin-2 pores were also observed to spontaneously form there. The definition of the conditions required for this observation opens the door to a much more detailed characterization of determinants of pore formation in the simulation of membrane-active peptides.

Our observation of AMP accumulation in the Ld phase supports the view that phase preference can potentiate AMP activity by promoting localized high-density peptide regions in the membrane (McHenry et al., 2012). Still, extrapolation of these conclusions to the much more complex bacterial or eukaryotic membranes, where sharp phase separation seems unlikely (Ingólfsson et al., 2014), should be done with caution.

## Data Availability Statement

The datasets generated from measurements on the performed simulations, on which analysis was performed, are included as a Supplementary Data archive.

## Author Contributions

JS performed simulations. SM and MM designed the research. MM performed the analysis. All authors wrote the manuscript.

## Conflict of Interest

The authors declare that the research was conducted in the absence of any commercial or financial relationships that could be construed as a potential conflict of interest.
