# Supplementary material for: Localization Preference of Antimicrobial Peptides on Liquid-Disordered Membrane Domains
Source: Front Cell Dev Biol. 2020 May 19;8:350. doi: 10.3389/fcell.2020.00350 (PMC7248343; doi:10.3389/fcell.2020.00350)
Supplement: Supplementary file 1 [file Data_Sheet_1.PDF]

Supplementary Material for

Localization Preference of Antimicrobial Peptides on Liquid-Disordered Membrane Domains

Juanjuan Su, Siewert J. Marrink, Manuel N. Melo

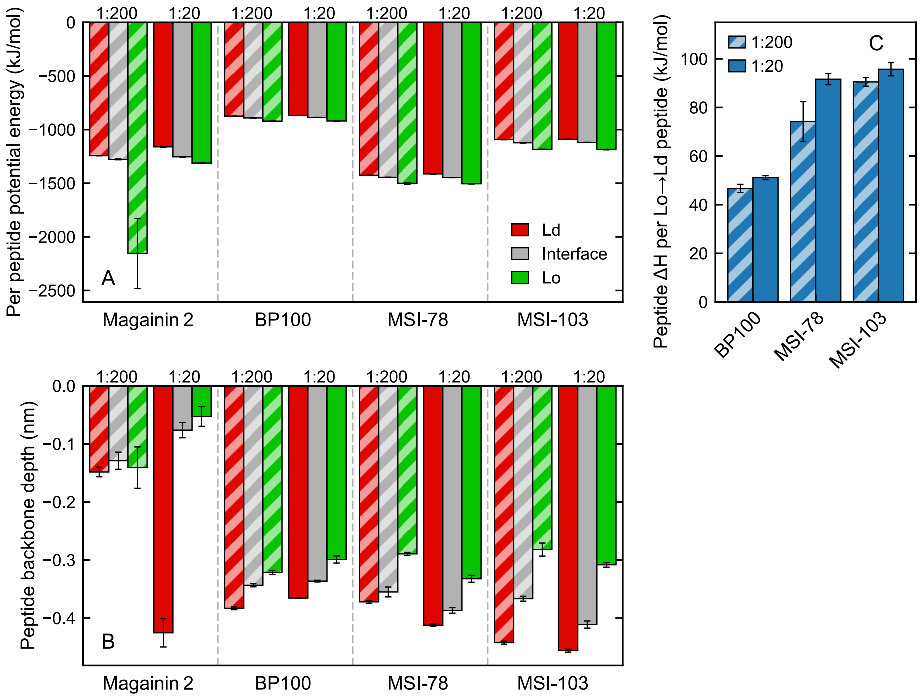

Supplementary Figure 1. Peptide interaction energies (A) and penetration depths (B) across domains, for both 1:200 and 1:20 P:L ratios of AMPs (as indicated on the top axis). C: Enthalpic cost, from the perspective only of peptide interactions, to move a peptide from the Lo to the Ld phase (corresponding to the subtraction of the Lo from the Ld energies in panel A). Lo energy and depth values for Magainin 2 at low concentrations are unreliable because the peptide was virtually absent from that phase during simulations, and it was therefore not included in panel C. The much lower average depth of Magainin 2 in the Ld phase at high concentrations reflects the formation of pores, with the concomitant movement of peptides towards the bilayer center.

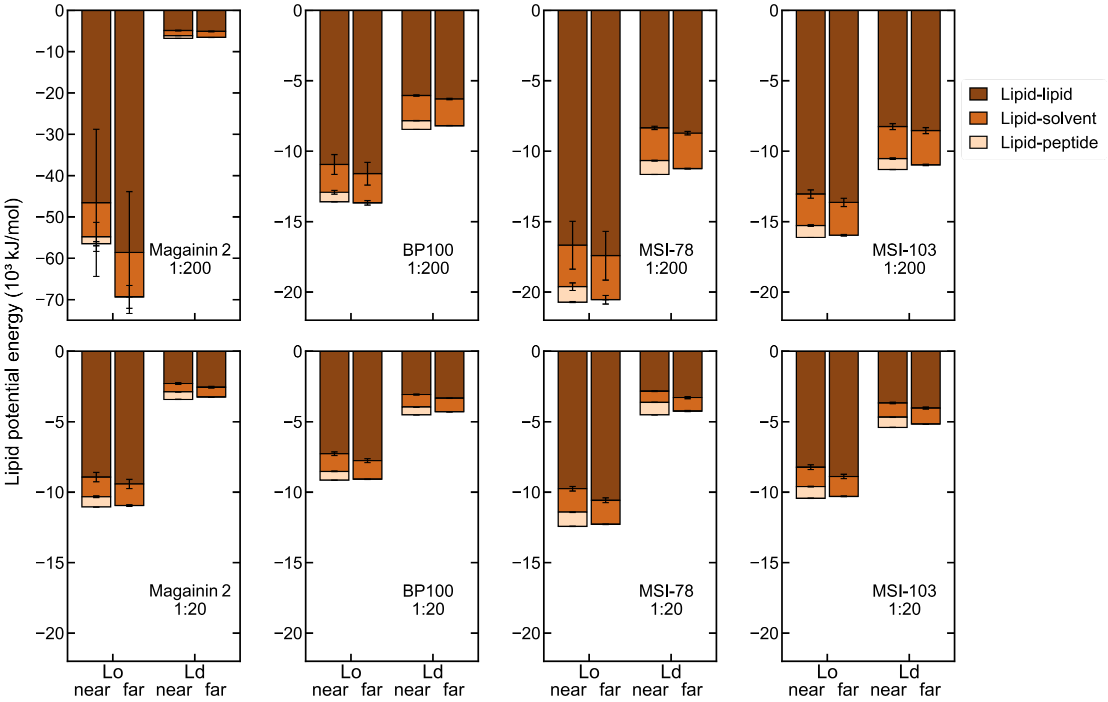

Supplementary Figure 2. Lipid interaction energies discriminated by proximity to peptides (defined as lipids with at least one particle within the 1.2 nm interaction cutoff of a peptide), lipid phase, and interacting molecule group (lipid, solvent, or peptides, as per the legend). Note the different scale for the top-left panel. Energies for lipids close to peptides are shown averaged by the number of peptides in that phase. To obtain a meaningful comparison, the energies of lipids far from peptides were averaged per lipid molecule type — DPPC, DLiPC, or cholesterol, for each phase. The average energies for each lipid types were then combined according to the numbers of each type close to peptides, in the respective phase; this aims to arrive at lipid energies that can be compared in the presence and absence of peptides (i.e. to assess how much the contacting lipids' energies change when a peptide is removed). This also involves normalizing by the number of peptides in each phase; because of this normalization, Magainin 2 values in the Lo phase — where there was very little peptide presence — can be seen to have uncertainties in the order of 10^4 kJ/mol, and are ill suited for estimating energy differences in the presence and absence of peptides. Note that because values are shown normalized per peptide, at higher concentrations (P:L 1:20) energies are of lower magnitude, reflecting the sharing of neighboring lipids by many peptides.

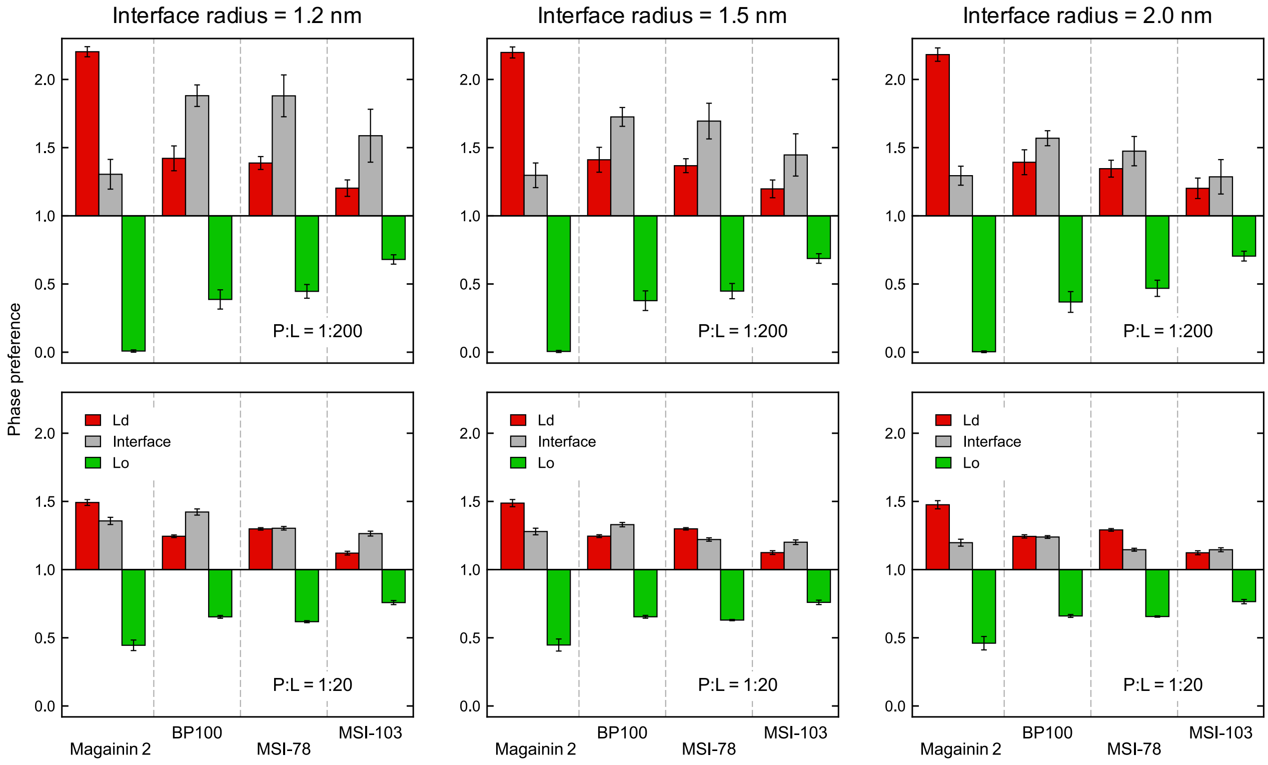

Supplementary Figure 3. Phase preferences analyzed at different radii (1.2 nm, 1.5 nm and 2.0 nm) for the definition of interface. The middle plots correspond to Figure 5 in the main text. Overall, at smaller radii, the interface becomes even more enriched, in relative terms. This likely reflects the inclusion of less area of the neighboring Lo region, typically depleted, in the definition of the interface.

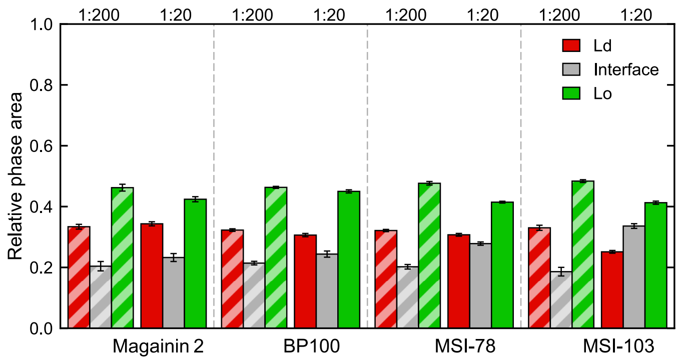

Supplementary Figure 4. Relative phase areas for the different simulated peptides and respective concentrations.

Functions for coulombic and Lennard-Jones potentials outside the GROMACS package

Simulations were carried out with standard Martini nonbonded interactions: a coulombic potential, $V_Q(r)$, with an implicit screening dielectric constant of 15, shifted to zero from r_1 = 0 nm to r_c = 1.2 nm; and a Lennard-Jones 6-12 potential, $V_{LJ}(r)$, shifted to zero between r_1 = 0.9 nm and r_c = 1.2 nm. Here r_1 specifies the onset of the shift function, and r_c the point by which the resulting potential becomes zero. These potential and shift functions are given, for interacting particles *i* and *j*, by:

$$V_Q(r) = f\frac{q_i q_j}{\varepsilon}V_1(r)$$ *Supplementary Equation 1*

$$V_{LJ}(r) = C_{12}V_{12}(r) - C_6 V_6(r)$$ *Supplementary Equation 2*

where r is the interparticle distance, q_i and q_j their respective charges, f the Coulomb constant, and $\varepsilon = 15$ the implicit screening dielectric constant. C_{12} and C_6 are multiplicative factors for the repulsive and dispersive components of the Lennard-Jones interactions, specified by the Martini forcefield for each pair of interacting particle types. The V_a functions V_1, V_6 and V_{12} are $1/r^a$ functions modified from r_1 to r_c so that they remain continuous and differentiable, and their value and derivative is zero at r_c. In GROMACS they take the form

$$V_\alpha(r) = \begin{cases} r^{-\alpha} - C & r \le r_1 \\ r^{-\alpha} - \frac{A}{3}(r-r_1)^3 + \frac{B}{4}(r-r_1)^4 - C & r > r_1 \end{cases}$$ *Supplementary Equation 3*

where A, B and C are constants that satisfy the imposed boundary conditions. They are defined as

$$A = \frac{-\alpha[3r_c + (\alpha+1)(r_c - r_1)]}{r_c^{(\alpha+2)}(r_c-r_1)^2}$$ *Supplementary Equation 4*

$$B = \frac{2r_c\alpha/(r_c-r_1) + \alpha(\alpha+1)}{r_c^{(\alpha+2)}(r_c-r_1)^2}$$ *Supplementary Equation 5*

$$C = \frac{1}{r_c^\alpha} - \frac{A}{3}(r_c - r_1)^3 - \frac{B}{4}(r_c - r_1)^4$$ *Supplementary Equation 6*

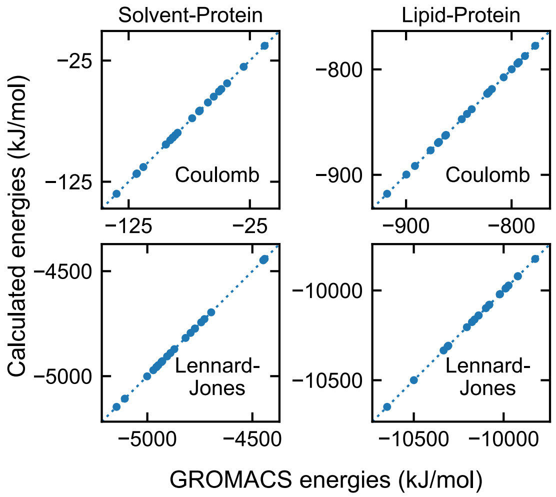

Supplementary Figure 5. Comparison of energies calculated by the GROMACS package for a 20-frame trajectory, and those calculated by the software developed for our phase-discriminated nonbonded potential energy analysis. Each data point corresponds to a frame, and the $x=y$ relation has been added as a dotted line. Each panel shows, for each frame, the sums of nonbonded interactions discriminated according to intervening groups (solvent vs. protein or lipid vs. protein) and type (coulombic or Lennard-Jones). This static energy discrimination is supported by GROMACS and can be mimicked by our energy calculation software by summing over the phase discrimination. The values are in close agreement, with a maximum absolute difference of 8×10^{-3} kJ/mol and a maximum relative difference of 3 ppm.
